# The self on its axis: a framework for understanding depression

**DOI:** 10.1038/s41398-022-01790-8

**Published:** 2022-01-18

**Authors:** Christopher G. Davey, Ben J. Harrison

**Affiliations:** Department of Psychiatry, The University of Melbourne, Melbourne, VIC Australia

**Keywords:** Depression, Neuroscience

## Abstract

The self is experienced differently in depression. It is infused with pervasive low mood, and structured by negative self-related thoughts. The concept of the self has been difficult to define—one of the reasons it is now infrequently an object of enquiry for psychiatry—but findings from functional brain imaging and other neuroscience studies have provided new insights. They have elucidated how the self is supported by complex, hierarchical brain processes. Bodily sensations rise through the spinal cord, brainstem, and subcortical regions through to cortical networks, with the default mode network sitting at the apex, integrating interoceptive signals with information about the extended social environment. We discuss how this forms a “self axis”, and demonstrate how this axis is set awry by depression. Our self-axis model of depression establishes a new perspective on the disorder. It emphasises the multi-level nature of depression, and how impacts made at different explanatory levels influence others along the axis. It suggests that diverse treatments might be effective for depression, from lifestyle interventions to psychotherapies to medications: they target different aspects of the self, but changes at one level of the self axis can affect others along it. Our framework for depression establishes a central role for the self, which might again become a useful focus of investigation.

The self changes in depression. It feels qualitatively different: it is a self that lacks volition, and that is infused with a sense of fatigue and aimlessness. The self is thought about in negative terms. The depressed person ruminates about the self’s deficits, feels guilty about past actions, and has a sense of foreboding and hopelessness about their future. When depressed, the way “we find ourselves in the world” is not the same [1].

Here, we explore the idea of the self as experienced differently in depression. We review concepts of the self, and how findings from brain imaging research have brought clarity. This research shows that internal models of the self map to brain networks that coordinate and integrate its diverse features, supporting the experience of a unified, singular self [2]. These processes are affected by depression, and we argue that viewing depression via a neuroscientifically informed understanding of the self provides a useful framework for understanding its symptomatic manifestations. It is a perspective that emphasises depression’s multifaceted nature, and the multiplicity of approaches that might be effective in treating it.

## The self defined

The self has fallen out of focus as an object of investigation in psychiatry. During the nineteenth and early twentieth centuries disintegration of the self was thought to be inherent in the development of psychotic disorders [3], and from the early to mid-twentieth century, psychodynamic theorists believed that disturbances of the ego (a concept not identical with the self, but close to it) explained depression [4, 5]. In recent decades, however, conceptualisations of depression have been dominated by focuses on the role of neurotransmitter systems, on the one hand, and irrational patterns of thought, on the other. While the latter includes distorted thoughts about the self, it is the negative character of the thoughts that have been the focus of attention. These theories of depression have tended to focus on only single explanatory levels. But as Kendler has argued, the complex nature of mental illnesses suggests they are much better understood as arising from the interaction of multiple levels [6].

The self has faded as a focus of study, in part because it is not clear what constitutes the object of investigation. What is the self? We have an intuitive grasp of what is meant by it, although it escapes easy definition [7]. There is, however, reasonably broad agreement as to its essential features: enough agreement to allow a sketch of the self that can be linked to underlying brain processes.

### The experiential and narrative selves

There are two basic aspects of the self. The first is the self that we experience in the present moment. It forms our subjective sense of being, and we apprehend its presence without need of focusing our attention on it. It has been described variously as the “core self” [8], the “basic self” [9], the “minimal self” [10], and the pre-reflective self [11]. We use the term “experiential self”—as suggested by Epstein [12] and Zahavi [13]—to highlight that this aspect of the self is known by direct experience without need for cognitive elaboration.

The experiential self is somatically embedded—influenced by the homoeostatic state of the body—and experienced in subjective, qualitative terms: in particular, as a self with an affective tone. It is this aspect of the experiential self that we argue is most pertinent to understanding depression. Damasio refers to the affective component of the experiential self as being composed of “background emotions” that he says provide “the feeling of life itself, the sense of being” [14]. These affective states, or moods, reflect the state of the body: influenced by processes such as states of wakefulness and satiety (or the relative lack of them), by illnesses and their accompanying inflammatory processes, and by discrete emotional experiences. They index the current state of the body as it finds itself in the world. Moods are internal, subjective experiences that cannot be reduced to more fundamental experiences. They might have external manifestations, but the mood state itself is private.

The second aspect of the self is that which becomes the object of our attention. This aspect of the self is composed of self, and self-other (i.e., social), representations. It has been referred to as the “empirical self” [15], the “autobiographical self” [8], and the “cognitive self” [12]. We use the term “narrative self”—as suggested by Dennett [16] and favoured by Gallagher [10]—to highlight that this is an aspect of the self that is perceived as having continuity over time, and that exists in a matrix of associations derived from experience. The narrative self builds on and extends the experiential self: we have a sense of the self as having existed in the past and we can envision its presence in the future. This is a sense of self that incorporates Cooley’s “looking-glass self” (a “looking glass” being an archaic term for a mirror): we think about how we appear to others and about how we are assessed by them [17]. We assume that others think about us in the same way we think about them, and have an internal representation of the shape such thoughts take. The narrative self has psychological content that is influenced by sociocultural factors: by the nature of the community in which we reside, both in the context of family and friendships and in the context of broader societal influences.

### The changing but singular self

Our sense of our selves is dynamic, varying with the nature of the representations presented to conscious awareness. It not only varies in content, but also to the extent that the self is represented. When we are focused on tasks, on events in the external world, we can sometimes have no awareness of the self at all (we “lose ourselves in our work”) [18, 19]. Similar experiences are reported by people who engage in deep meditation or in psychedelic drug experiences [20, 21]. In depression the converse is often true: the depressed person can become so absorbed with internal processes that they find it difficult to focus on events outside of them [22].

An important feature of the self is that it is perceived as a unitary phenomenon. While we emphasise two aspects of the self—experiential and narrative—they are experienced as one, and are in fact not cleanly delineated, but exist at either ends of a continuum [8]. The sense of self can vary in different contexts and during different periods of life (including during depression), and can sometimes feel more fragmented and at others more cohesive, which might suggest we contain multiple and divided selves. The self as we have defined it, however, while complex and changeable, is *experienced* as singular: in part because the self is intimately related to a single body [18, 23]. It has a unitary character that we experience as being at some central part of our conscious awareness, as captured by Strawson’s description of the self as an “internal mental presence” [24], and Dennett’s as “the center of narrative gravity” [16].

In summary, the essential characteristics of the self as we have defined it:

1. The self is apprehended as a unitary phenomenon, although it consists of different aspects.
2. One aspect — the experiential self — forms our fundamental sense of being. It is embedded in our body, and is imbued by the mood states that arise from somatic processes.
3. The other aspect — the narrative self — is the self that we can think about, and that we have reason to believe other people think about in the same way that we think about them.
4. These aspects exist at either ends of a continuum, and co-exist at any particular time to a greater or lesser extent.
5. The self varies in its presentation, with different aspects present in different contexts, which might suggest that it doesn’t really exist at all. But to most of us, the self exists as a matter of everyday experience.

## The self in depression

Depression has a profound effect on a person’s subjective sense of being-in-the-world. It is not simply the same self as it was before, but with symptoms of depression superimposed on it. It is now a *different self*, inhabiting a world that is perceived in a different way [1]. It is a self that is instilled with a pervasive low mood, inhabiting a world that lacks possibility for positive experiences, and in which there is in any case no energy or motivation to pursue positive experiences.

The symptoms that comprise the diagnosis of major depressive disorder can be usefully categorised into symptoms that reflect affect and drive (depressed mood, anhedonia, and reduced motivation), that have cognitive manifestations (self-criticism, concentration difficulties, and suicidal thoughts), and that reflect basic life processes (changes in sleep, appetite, and psychomotor activity). These symptoms can be present in many combinations, and in the case of somatic processes can be of opposite polarities, such that there are argued to be multiple types of depression. Efforts to categorise depression over the past century—melancholic versus non-melancholic, typical versus atypical—have not proven to be clinically useful [25, 26]; in part because the symptoms do not segregate in consistent ways [27]. More recent approaches have examined the symptoms of depression using network analyses, demonstrating how the presence of particular symptoms seems to drive the development of others, which in combination with other symptoms influence yet others in patterns that are complex and variable. While these analyses confirm that symptomatic patterns do not form readily identifiable subtypes, they show that some symptoms are more fundamental than others; and central to depression is depressed mood [28, 29].

### Depressed mood, disrupted somatic functions, and the self

The self in depression is imbued by depressed mood, which colours all aspects of a person’s experience of themselves. Depressed mood is, like the self, difficult to define, with one of its very characteristics its seeming ineffability. As the writer William Styron describes it: “Depression is a disorder of mood, so mysteriously painful and elusive in the way it becomes known to the self—to the mediating intellect—as to verge close to being beyond description” [30]. It is often described in analogical and metaphorical terms: for example, as like inhabiting a “bleak shadowland” [31]; or a “darkness … closing in” [32]; or “some kind of hell, containing nothing from which [one] could obtain relief or comfort” [33].

Depressed mood is an emergent property of processes related to the somatic state. It is a concomitant of the alterations in sleep, appetite, psychomotor activity, energy levels, sex drive, gastrointestinal function, and pain sensitivity that are characteristic of depression. It can arise as a result of physical illness: the low mood that often accompanies a short-lived viral infection is familiar to most of us [34]. Depressed mood shapes the experiential self to give it its qualitative attributes, and by doing so influences cognitive representations of the self. As we will discuss, the brain regions that instantiate the core aspects of the self send descending projections to the hypothalamus and brainstem regions that regulate sleep, appetite, and other drives. These basic life functions therefore not only shape the experiential self, but are influenced by the very brain processes that give rise to it. The recursive relationship between these basic drives and the self is indicative of the deeper homoeostatic functions the drives subserve, and highlights how the enduring function of the self is to promote the maintenance of homoeostatic processes in the face of environmental demands (i.e., allostasis). Understanding depression as a response to “allostatic load” [35], manifested by disturbance in basic life functions, inherently involves a change in the way the self is experienced.

### Negative views of the self in depression

Depression affects the way people think about themselves, with the self characteristically seen in a negative light—in the present and across time. The depressed person sees themselves, says Beck, as “defective, inadequate, diseased, or deprived”, and “tends to attribute [his or her] unpleasant experiences to a psychological, moral, or physical defect” [36]. Not only does the depressed person think about themselves in more negative terms, but they are also more sensitive to indicators that support their views: for example, by selectively focusing on negative feedback [37] and by recalling more negative self-descriptors [38].

The way the self is understood in the context of relationships also changes with depression. The depressed person believes that they place a burden on people within their social networks, no longer feels the same sense of belonging to them, and finds social events different to enjoy [39]. They respond by withdrawing from social interactions, and their isolation reinforces their negative beliefs about themselves.

A commonly observed symptom in depression is an increase in self-focused attention [40]. People with depression spend more time thinking about themselves, and can find it difficult to switch their attention to events outside of themselves [22, 41]. Once they have switched their attention to external events, they often find it difficult to maintain their concentration. They more readily revert to self-related thoughts, manifesting as distressing rumination. We might understand that the low mood that permeates the self draws attention to internal processes. Such negatively valenced affective processes not only capture attention, but also imbue narrative conceptions of the self with negative content, which further focuses attention on the self. That the self is experienced differently in depression has long been clear, but the brain mechanisms that might help to explain it have been less so.

## A brain model of the self

The self might not exist in the same way that the heart or spleen exists [39], but it is nonetheless a dynamic entity that is closely tied to underlying brain processes. Mapping the self to such processes has been facilitated by the development of brain imaging techniques, and especially functional magnetic resonance imaging (fMRI). These methodologies have led to the identification of distinct brain activity signatures that support the generation of internal mental experiences, arguably one of the most original and significant achievements of human neuroimaging research.

### The default mode network and the self

The brain regions that have come to be most identified with the self comprise the default mode network (DMN), which incorporates midline medial prefrontal cortex (MPFC) and posterior cingulate cortex (PCC), and well as the inferior parietal lobules and other regions. The DMN was first described in early nuclear imaging studies, when these regions were found to display a unique pattern of activity that was more pronounced during passive mental states, such as wakeful rest, than during goal-directed cognitive tasks [42]. The concept of a “default mode” conveyed the idea that this was a pattern of brain activity that was consistently suppressed during task performance—when one’s attention was externally focused—and was defaulted back to in the absence of such task demands [43]. It was hypothesised that DMN regions—especially the medial frontal cortex—supported the introspective, self-oriented mental processes that are characteristic of resting state conditions [43, 44]. The link between DMN function and resting mental activity has since been extensively characterised [45–48].

The idea that DMN activity underpins self-related processes has been supported by task-based fMRI studies. The tasks have directly addressed self-referential thought [49–51], and have also assessed autobiographical memory and future forecasting [52, 53], moral decision making [54, 55], and theory of mind [56, 57]. They have provided some of the clearest evidence of the DMN’s involvement in functions related to the self. Importantly, across these different task domains only a subset of DMN regions are commonly engaged. That is, while DMN activity has been anatomically characterised in each of the aforementioned task domains, there exist a smaller “core” set of regions that support self-related processes in a more domain-general manner. Although the precise computations supported by these core regions remain to be fully understood, it has been suggested that they might support basic self-oriented evaluations of the significance, value, and meaning of stimuli [52, 54].

In our own work we employed a task designed to isolate the activity of core DMN regions during the appraisal of personality traits, and used a network modelling approach to determine their dynamic interactions [49]. We found that self-appraisal was primarily co-ordinated by the PCC, with its activity modulated by regulatory feedback from the MPFC. In functional terms, we suggested that narrative self-representations, such as those engaged during the semantic processing of personality trait adjectives, were dynamically accessed via PCC (through its dense connections with posterior and temporal brain systems) and gated into conscious awareness by activity in the MPFC. This hypothesised gateway function of the core MPFC region aligns with its location in frontopolar cortex (Brodmann Area 10). The regions appear to coordinate competing internal and external representations, including self-representations, to guide ongoing thought processes and behaviour [58]. We argued that it was via these dynamic interactions that a unitary sense of self emerged.

This conception of the core DMN as a “self-making” network is reinforced by findings that these regions are among the brain’s most highly integrated [59]. Recent characterisations of the DMN’s connectional anatomy has recognised its position at the apex of a deep hierarchy of brain systems; existing at one end of a spectrum, which starts with primary sensory and motor cortices at the other [60, 61]. Intermediate to these are the sensory and motor association areas, and other association networks that are involved with conceptual organisation of information related to environmental input, such as the central executive and salience-interoceptive networks. Being the least tethered of association networks to inputs from the external environment, the DMN appears to act as a master integrator of representational content across other brain systems [62, 63], providing it with a unique capacity for integrated information processing.

We have argued that the DMN’s role in flexibly integrating representations across conceptual space—from concrete sensory representations to more abstract schematic representations—are fundamental to its role in representing the self [64]. Recent studies have emphasised a more general role for the DMN in human cognition, characterising it as supporting high level sense-making and conceptualisation [65, 66]. Such roles are compatible with the self-making hypothesis, which incorporates elements of both. While the integrative functions of core DMN regions have been primarily studied in the domain of narrative self-related processes, a more compelling model emerges when considering the role of core DMN regions in supporting integrative processing across the narrative and experiential self domains, “instantiating a uniquely deep and domain-general model of the embodied [self]” [67].

### The DMN and the experiential self

The DMN’s integration of information includes interoceptive representations of the state of the body, which form a core part of the experiential self. The main cortical contributor to the representation of interoceptive states is the anterior insula. It also sits atop a hierarchy of neural processes that extend from the peripheral nervous system, to brainstem and subcortical regions, comprising the brain’s central autonomic (or salience-interoceptive) network. The higher-order representations that are encoded by the anterior insula are integrated with other cortical networks to provide the somatic component of self-representations [68]. This integration with core regions of the DMN is facilitated by distinct subcortical integrative zones in the basal ganglia and thalamus [69].

The peripheral nervous system is continuous with the body. The afferent pathways relevant to interoception are composed largely of type C unmyelinated fibres, which are exposed to the extracellular environment along the lengths of their axons [70]. These phylogenetically ancient systems provide information on the internal milieu, and help to generate the positive and negative feeling states related to basic life functions (e.g., thirst, hunger, pain), and basic emotions (e.g., sadness, fear, joy).

Interoceptive pathways interact with subcortical-prefrontal systems that coordinate the affectively valenced character of mood-states: these comprise the fronto-amygdala, frontostriatal, and hippocampal-prefrontal circuits. They have in common that the subcortical regions send efferent projections to the MPFC, which acts to integrate the signals and provide top-down regulation of the processes [71–73]. The subcortical-prefrontal circuits are modulated by monoaminergic systems, which also modify the mood-state. The MPFC is richly innervated by serotonergic, noradrenergic, and dopaminergic neurones; as are the amygdala, striatum, and hippocampus [74, 75]. The MPFC in turn projects to monoaminergic neurons. It is the main source of afferent connections to the dorsal raphe nucleus, from where most serotonergic projections originate [76].

The experiential self, imbued with feelings and motivations, is underpinned by the complex interplay between the interoceptive, subcortical-prefrontal, and monoaminergic systems. These systems are represented at higher levels via activity of the anterior insula and medial cortical regions, and via these regions and their cortical interactions influence the content of the narrative self.

### The hypothalamus and basic life functions

The relationship between brain processes supporting the experiential self and bodily processes is recursive. MPFC and subcortical regions send efferent projections to the hypothalamus and other regions important for basic life processes, including the periaqueductal grey (PAG), parabrachial nucleus, and the dorsal raphe nucleus [76–78]. The hypothalamus is composed of a number of specific circuits, which, together with mid-brain and brainstem regions, have a key role in activating autonomic, endocrine, and behavioural responses [77]. The activity of these circuits influences approach and withdrawal behaviours, and regulates basic functions such as sleep, appetite, sexual drive, and the stress response. These functions effect the internal milieu, which in turn contribute to the mood state and experiential self.

### The self axis

As a matter of experience the self is bound into a single phenomenon: one that can be felt subjectively, and at the same time thought about as a cognitive entity. Here we might consider the complex, hierarchical, integrated brain processes that support the sense of self as forming an axis. From a brain perspective, the system commences with the processing of bodily sensations at the level of the spinal cord, rising through brainstem and subcortical regions, and then to cortical networks that integrate contextual information, with the DMN and its core regions sitting at the apex. Activity across these levels coincides with a unified experience of self that spans the continuum from subjective felt experience to abstract self-representations. The self axis therefore encompasses both the brain processes and their psychological correlates, consistent with a dual-aspect conception of material and mental processes [79, 80].

As we have emphasised, the MPFC has a particularly important role [49, 64]. It operates as an intermediary between external and internal representations, as befits its rich club role in integrating cortical network activity. As our own work has shown, MPFC has a gating effect on PCC activity, selecting relevant self-representations as appropriate to the external context [49]. The MPFC also acts as the fulcrum between the cortical and somatically embedded brain systems. It is not only a key contributor to cortical network coordination, but also directly influences autonomic and neuroendocrine output. It generates “affective meaning” by integrating affective processes with the relevant environmental context [81].

**Fig. 1:**
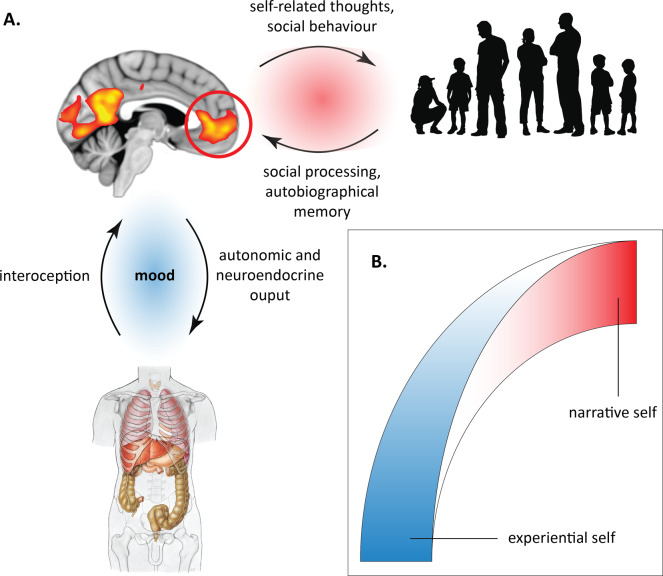
The self axis. The self is a dynamical entity that emerges from reciprocal brain relationships with the body and social environment (**A**). The self exists on a continuum from a subjective, pre-reflective “experiential” self to an objective, cognitively represented “narrative self”: forming what we term the self axis (**B**). The medial prefrontal cortex (MPFC; circled), a part of the default mode network, has a key coordinating role, intermediating between apprehension of the social environment and somatic processes. Mood emerges from somatic processes, under top-down influence of the MPFC. It forms a fundamental part of the experiential aspect of the self, both influencing and being influenced by the narrative aspect.

Here we can understand that the MPFC and PCC are highly active brain hubs that are continually adjusting activity along the self axis and generating a unitary sense of self [64]. The self is dynamic, changing from moment to moment in accordance with environmental demands, both external and internally generated. These different experiences of the self are influenced by particular interpersonal contexts, and by somatic influences mediated by the mood-state (Fig. 1).

### The self as generative model

Recent conceptualisions of the brain suggests it actively constructs high-level generative models that predict the source of sensory inputs at lower levels, adjusting the models (or the sensory data sources) in response to ascending sensory error signals in the service of promoting adaptive behaviours [82]. In this framework, the experiential self models the interoceptive environment, with mood arising as the manifestation of error signals that influence behaviour and attention, as succinctly described by others [83, 84]. But our model of the self expands on this conception of the self to include its narrative features. This enriched self sits at a deeper level of the predictive hierarchy, incorporating a model of interoceptive processes, but broadening it to contextualise it within the social environment. The DMN coordinates the construction of this generative model, which can be summarised as forming an apperception of a person’s embodied presence in the world. The model is constantly readjusted as it infers the causes of internal and external (mainly social) sensory inputs, but has a consistency borne of its integration with a singular body, which is modelled interoceptively and projected into the social world (in the present, in memory, and in the hypothesised future).

## The self axis in depression

The brain regions that are implicated in depression show considerable overlap with the regions that support the self, with multiple levels of the self axis being affected, modulated by the activity of neurotransmitter and neuroendocrine systems [85, 86].

### Cortical and subcortical influences

The significant role that the MPFC plays in depression has been one of the most striking finding from imaging studies of the disorder. Early nuclear imaging studies showed that depression was characterised by increased activity of the subgenual cingulate cortex [87–89], a part of ventral MPFC; and other studies have confirmed increased resting-state activity and connectivity with the region [90, 91].

Studies that have examined how thinking about the self’s attributes affects MPFC activity have been inconsistent, showing both increased [92, 93] and decreased activity [94, 95], in part related to the different nature of the tasks (e.g., responding to pictures versus words). Our own study examined the brain response of depressed patients as they judged whether self-descriptors applied to them, assessing effective connectivity between brain regions activated by the task (i.e., the influence that one brain region had on another). It showed that MPFC exerted greater negative influence on the PCC while depressed participants thought about themselves than it did in healthy control participants [96]. The extent of this was related to a symptom factor associated with difficulties with attention, and was also stronger when participants had social anxiety disorder, leading us to surmise that the more negative MPFC-PCC connectivity observed in depressed patients reflected their increase in self-focused attention, and difficulties in shifting their attention from internal to external representations.

Difficulties with attention are also reflected in a reduction in activity and connectivity of the dorsolateral prefrontal cortex in depressed participants [97, 98]. Dorsolateral and medial cortical regions have been observed to show anti-correlated activity in normal brain function [99, 100], and in depression the reduction observed in dorsolateral prefrontal cortical activity is correlated with increased subgenual cingulate cortex activity [101]. These shifts in balance are also manifested as a relative failure of depressed patients to suppress MPFC activity during attentionally demanding tasks, suggesting an intrusion of self-related thoughts during efforts to concentrate on events external to the self [102]. Altered activity in subcortical regions contribute to the experience of the self in depression, with increased amygdala responsivity to emotional faces [103, 104] suggesting increased sensitivity to the social environment; and decreased activation of the striatum to self-related stimuli [94] and to rewards [105], together with its reduced connectivity with MPFC [91, 106], contributing to anhedonia.

### Neurotransmitter systems

There is longstanding evidence that monoaminergic systems are implicated in depression, with most evidence for serotonin [107]. However, studies that have shown abnormal plasma and cerebrospinal fluid concentrations of serotonin, and altered levels of serotonin receptors and transporters, have been inconsistent [108]. Perhaps the best evidence for serotonin’s involvement in depression is that depressive symptoms can be improved by medications that affect the serotonergic system, particularly by blocking its presynaptic reuptake. The effects of selective serotonin reuptake inhibitors are, however, inconsistent at an individual level, with only some patients gaining benefit [109].

There has been more recent focus on the roles of other neurotransmitter systems in depression, and particularly on the brain’s primary excitatory neurotransmitter, glutamate. Glutamate levels in the MPFC are reduced in depression [110, 111], and other abnormalities in the glutamatergic system are also evident [112], likely reflecting the observed changes in cortical and subcortical network connectivity. Interest in the role of glutamate in depression has been spurred by evidence that agents that act on the system, such as ketamine, have antidepressant properties [113, 114]. It has become more evident that our early understanding of depression as arising from dysfunction of the serotonergic system was overly simplistic; and that depression is in fact unlikely to be explained by altered function of any single neurotransmitter system.

### Neuroendocrine and autonomic nervous system function

The neuroendocrine and autonomic nervous systems enact the influence of brain processes on the body, and alterations in their activity are reflected in depressive symptoms such as lethargy and insomnia that contribute to the mood state. The hypothalamus has a central role in these processes. It receives extensive afferent connections from the MPFC, and sits itself atop the hypothalamic-pituitary-adrenal (HPA) axis. Many studies have found that the HPA axis is hyperactive in depressed patients, who show increased cortisol levels [115, 116] and aberrant response to feedback, with hypothalamic corticotrophin-releasing hormone secretion not decreasing in the face of hypercortisolaemia [117]. These processes have important influences on the inflammatory response, and a number of inflammatory parameters are altered in depressed patients [118]. The hypothalamus has a controlling influence on sleep and appetite, partly mediated via the HPA axis, but also by discrete hypothalamic circuits (e.g., the lateral hypothalamic orexin system [119]), which show some evidence of disturbance in depression [120, 121].

Autonomic functioning is disturbed in depression. Heart rate variability, a measure of the fluctuation in the interval between heart beats, is reduced, reflecting change in the balance between sympathetic and parasympathetic activity [122]. Depressed patients commonly complain of gastrointestinal symptoms such as bloating and cramping, which likely reflect disturbances in gut motility and function, which are also under the control of the autonomic nervous system [123, 124].

Together, these alterations in the brain’s influence on bodily functions contribute to the characteristics of depressed mood, generating many of its phenomenological features. The feelings that arise from fatigue, anergia, insomnia, anorexia, and low-level inflammation affect the foundations of the self as it is experienced in depression. The feelings are elaborated in thoughts that condemn the self as unworthy and guilty and in behaviours that lead to social withdrawal and isolation. These experiences of the self, underpinned by alterations in brain activity and connectivity in cortical and subcortical brain regions, influence hypothalamic function and physiological processes. The depressed self is tilted off its axis, establishing itself in a new state in which multiple levels of the self have been set askew.

## Reconceptualising depression and its treatment

Our self axis model of depression establishes a new perspective on the disorder. It emphasises the multi-level nature of depression, and how different explanatory levels influence others along the axis. When depression develops, the original disturbance can occur at any one of multiple levels of the self axis, but propagates along it. For example, a young man’s depression might arise from a relationship disappointment, in which the way he views himself changes: he now sees himself as being unlovable, lonely, and disconnected. These representations of the self are associated with an increase in self-directed thoughts, manifested as distressing rumination. There is an increase in autonomic nervous system activity, mediated by MPFC, with anxiety and somatic preoccupation exacerbating his rumination. Hypothalamic dysfunction, also under top-down influence of the MPFC, is reflected in insomnia and fatigue, and the syndromic manifestation of major depressive disorder emerges.

These symptoms might also arise without any evident precipitating factor, as is common. A middle-aged woman reports an incipient increase in sleeping difficulties, feels more fatigued, and is not able to find pleasure in things. These negative feelings lead to an inward focus on internal processes, mediated by a shift in medial versus dorsolateral frontal cortical activity. She ruminates, and her negative affect imbues thoughts of herself as unlikeable and ineffective. Her mood deteriorates further, and she begins to struggle in her social and work roles, further entrenching her negative views of herself. While her depression did not begin with an obvious psychosocial stressor, her symptoms of depression are similar to the young man’s.

In the predictive processing framework, these cases of depression can be viewed as discrepant responses to error signals that first arise in the social and interoceptive environments, respectively. A number of theories based on predictive hierarchical models have been put forward to explain how depression arises from maladaptive inferences in response to such error signals [125–127], although the theories are partial, explaining only particular aspects of depression, and remain tentative.

In both vignettes, negative thoughts are features of the depression. In neither case did depression start with those thoughts (although in other cases they might). Within our framework of understanding depression via the lens of the self, elaborated by a deeper understanding of its neural underpinnings, negative thoughts—including negative thoughts about the self—are a symptom of depression but do not have a privileged status. They are manifestations of perturbations of the self axis, and a focus on dysfunctional thinking to the exclusion of other factors over-emphasises their role. That does not mean that a therapy that focuses on negative thoughts might not be an effective treatment for depression. If we consider that depression is the manifestation of a self axis that has been set awry, then any treatment that targets one of the components of the axis might correct deviations along it, without it having to target the level that was first perturbed in the person’s depression. And negative thoughts present a particularly useful lever—one that is available as a treatment target and that is amenable to correction via cognitive behavioural therapy (CBT).

Our view of the self in depression also suggests other targets. Lifestyle initiatives that encourage better physical health—healthy eating, exercise, and sleep interventions—can improve mood by directly affecting the physical parameters that affect the mood state and set the foundations of the experiential self. Such interventions require behavioural changes, which can also require clinicians encouraging patients to acquire a different perspective on their narrative selves: as being someone who is capable of making changes, and whose efforts will not be futile. Many interventions—even one as seemingly straight forward as a dietary intervention—target multiple levels of the self axis. Psychotherapeutic approaches can emphasise aspects of the self other than the nature of the thoughts a patient has about themselves. They can focus on the self as it experienced in the context of relationships, including the therapeutic relationship. The depressed person can be encouraged to spend more time engaging with people who have been important to them, thereby helping to reintroduce a sense of a self that is connected with and valued by others.

In our view of the self as spanning multiple explanatory levels and as having both brain and psychological aspects, the use of medications is not antithetical to other treatment approaches. As we have discussed, the brain processes that support the self are set awry by depression, and it is possible that monoaminergic, glutamatergic, and other medications are able to influence brain activity and connectivity in ways that shift the self axis’s deviation. As with the targeting of negative thoughts with CBT, the use of a medication that affects a particular neurotransmitter system does not imply that the depression was caused by dysfunction of that system. But the neurotransmitter system might provide a target that can be used to influence perturbations across the self axis. Neurostimulation treatments such as transcranial magnetic stimulation can similarly influence network activity—targeting the dorsolateral prefrontal cortex to increase its activity can result in correction of the increased subgenual cingulate cortex activity that is often observed in depression [128, 129]—but the effectiveness of the treatment does not imply that depression is caused by these changes in network activity and connectivity.

Our brain architecture of the self describes a complex, hierarchical system, that spans neuronal to psychosocial processes. There are many ways in which the system might be affected to manifest depression, which presents clinically with such a diverse range of symptoms. Treatments for depression have only modest overall effectiveness: many people do not respond to particular treatments, whether they be CBT, antidepressant medications, or exercise interventions. As a field, we have had difficulty identifying the characteristics of a person’s depression that might make it more responsive to one treatment or another. Our model emphasises the plurality of explanations for depression, and the plurality of treatments that are often needed to treat it.

## Conclusion

We conceptualise the self as a dynamic entity whose psychological aspects mirror their underlying brain processes, extending across an axis that reaches from the body to the social environment. The mood-state indexes the state of the body as it finds itself in the world, and in doing so provides a foundational sense of self. It manifests the physiological state of the body, which is responsive to the social context, mediated by the activity of association networks that are coordinated by highly connected hubs within the core DMN. Mental representations of the self thus incorporate multiple levels, including feeling states that emerge from the body, and representations of the self as it exists within an interpersonal matrix.

This way of understanding the self provides us with a framework for understanding depression. Depression is first and foremost a subjective experience, and one that affects the very core of a person’s sense of themselves. By taking our concept of the self as an organising principle for depression, we can integrate multiple explanatory perspectives, adopting the sort of multi-level pluralism that has been argued as necessary for understanding mental disorders [6]. In this framework the depressed person does not disappear in the scientific gaze to be replaced by neurotransmitters and cognitions, but remains prominent and centred within it.
